# GhABF2, a bZIP transcription factor, confers drought and salinity tolerance in cotton (*Gossypium hirsutum* L.)

**DOI:** 10.1038/srep35040

**Published:** 2016-10-07

**Authors:** Chengzhen Liang, Zhaohong Meng, Zhigang Meng, Waqas Malik, Rong Yan, Khin Myat Lwin, Fazhuang Lin, Yuan Wang, Guoqing Sun, Tao Zhou, Tao Zhu, Jianying Li, Shuangxia Jin, Sandui Guo, Rui Zhang

**Affiliations:** 1 Biotechnology Research Institute, Chinese Academy of Agricultural Sciences, Beijing 100081, China; 2 Department of Plant Breeding and Genetics, Bahauddin Zakariya University, Multan, Pakistan; 3 College of Agronomy and Biotechnology, Southwest University, Chongqing 400715, China; 4 Biotechnology Research Department, Ministry of Science and Technology, Naypyidaw, Myanmar; 5 National Key Laboratory of Crop Genetic Improvement, Huazhong Agricultural University, Wuhan, Hubei 430070, China

## Abstract

The bZIP transcription factor (TF) act as an important regulator for the abscisic acid (ABA) mediated abiotic stresses signaling pathways in plants. Here, we reported the cloning and characterization of *GhABF2*, encoding for typical cotton bZIP TF. Overexpression of *GhABF2* significantly improved drought and salt stress tolerance both in *Arabidopsis* and cotton. However, silencing of *GhABF2* made transgenic cotton sensitive to PEG osmotic and salt stress. Expression of *GhABF2* was induced by drought and ABA treatments but repressed by high salinity. Transcriptome analysis indicated that *GhABF2* increases drought and salt tolerance by regulating genes related to ABA, drought and salt response. The proline contents, activity of superoxide dismutase (SOD) and catalase (CAT) were also significantly increased in *GhABF2*-overexpression cottons in comparison to wild type after drought and salt treatment. Further, an increase in fiber yield under drought and saline-alkali wetland exhibited the important role of *GhABF2* in enhancing the drought and salt tolerance in transgenic lines. In conclusion, manipulation of GhABF2 by biotechnological tools could be a sustainable strategy to deploy drought and salt tolerance in cotton.

Drought and salinity are main limiting factor for plant growth and productivity^1^,^2^. Plants have well developed sophisticated signaling pathways *i.e*. receptors, secondary messengers, phytohormones, and signal transducers for their survival in stressed and complex environment^3^,^4^. The response of plants against abiotic stresses also required differential gene expression mainly regulated by Transcription factors (TFs). The TFs are key components in controlling transcription initiation rates to modulate a series of stress-associated genes expression for the abiotic stress signal cascade transmission^5^,^6^,^7^. The genes expression regulation is responsible for synthesis, degradation of specific proteins, enzymes, metabolites those together constitute the defense response against biotic and abiotic stresses^8^. Therefore, TFs are pivotal for plant scientists to improve the tolerance against various stresses^3^.

The bZIP (basic leucine zipper) protein is one of the largest TF families in plants. They are characterized by conserved bZIP motif having basic region responsible for specific DNA-binding coupled with a leucine zipper required for TF dimerization^9^. About 75 bZIPs TFs in *Arabidopsis*, 92 in rice, and 89 in *Populus trichocarpa* have been identified^9^,^10^,^11^. According to DNA binding specificity and sequence similarities of bZIP domain, these bZIP TFs can be divided into 13 groups (A, B, C, D, E, F, G, H, I, J, K, L, and S)^9^. Recent work revealed that group A of bZIP TFs, includes ABI5^12^, ABF1^13^, ABF2/AREB1^14^, ABF3^15^, and ABF4^16^ of *Arabidopsis thaliana*, involved in ABA-regulated and stress-induced gene expression^17^. In rice the Group A TFs TRAB1^18^, OsbZIP23^19^, OsABF1^20^, and OsABI5^21^ also play an important role in ABA signal transduction and osmotic stress responses. In addition, the bZIP Group A TFs have been shown to form heterodimers in some combinations and tolerate variability in the ACGT core element essential to the abscisic acid (ABA)-responsive elements (ABRE) G-box^17^,^22^, indicated that they may participate in the regulation of same target genes and ABA-mediated stresses response signaling pathways. Thus, conservation of the Group A bZIP TFs transcriptional regulatory system for ABA-mediated abiotic stress response has been proposed.

Cotton (*Gossypium hirsutum* L.) is one of the most economically important crops and provides natural fiber to textile industry around the globe^23^,^24^. Although cotton has moderate amount of tolerance against drought and salinity but its growth, yield, and fiber quality are effected by the severe environment conditions^25^. Furthermore, increasing competition for arable land between food and cash crops, development of genetically engineered stress tolerant cotton genotypes for marginal land, such as the low beaches and saline-alkali land, at the eastern coast and northwest region of China could yield encouraging results. To achieve this target, cloning and characterization of key target genes those can contributes in enhancing cotton stress tolerance could be a modern prospective agricultural approach. The success of herbicide tolerance and Bt (*Bacillus thuringiensis*) cotton have paved the way for development of transgenic cotton having drought and salt tolerance^26^,^27^,^28^. Expression of several potential stresses-related candidate genes including *AtEDT1/HDG11*^25^, *AtNHX1*^29^, *AVP1*^30^, *TsVP *31, *SNAC1*^32^, *IPT *33, and *AhCMO*^34^ from *Arabidopsis*, *Thellungiella halophila*, rice, tobacco, *Escherichia coli (E. coli)*, and *Atriplex hortensis* contributed significantly to enhance drought and salt tolerance in cotton. Recently, several transcriptome profiling have been employed to identify stresses responsive genes in cotton^35^,^36^,^37^,^38^,^39^,^40^,^41^. In these microarray platforms, members of Group A bZIP TFs were strongly induced by single or multiple stresses. Based on these results we can reasonably hypothesize that Group A bZIP TF are closely related with enhanced stresses tolerance in cotton. Keeping in view this scenario, we demonstrated that a new member of Group A of bZIP TF, GhABF2, can play a key role in controlling ABA-mediated drought and salt tolerance in cotton. The characterization of *GhABF2* will further deepen the understanding to the regulatory mechanism underlying stress tolerance, and facilitate the rational applications for development of drought and salt tolerance cultivars.

## Results

### Identification of bZIP transcription factor GhABF2

**Figure 1:**
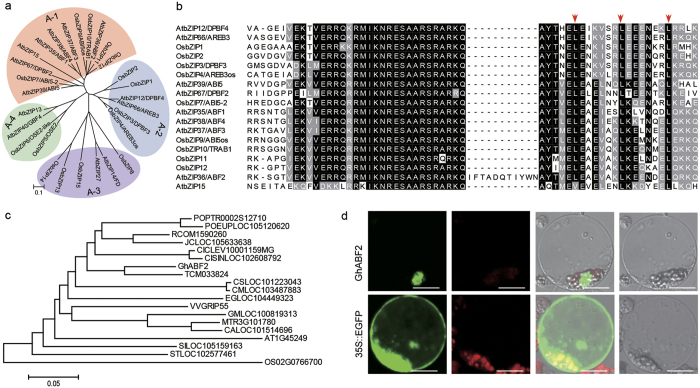
Cloning and characterization of *GhABF2* and subcellular localization of the GhABF2-GFP fusion protein. (**a**) Phylogenetic analysis of Group A bZIP TFs in *Arabidopsis* and rice. The scale bar indicates 0.1 amino acid substitution per site. (**b**) Comparison of conserved bZIP domains of Group A TFs. Sequences were aligned using ClustalX. Conserved amino acids are highlighted. White letter on black background highlights those amino acids conserved across all of Group A samples (100% conservation), while black letter on gray background highlights amino acids conserved in 60% conservation of the samples. Red arrows indicated the Leucin. (**c**) Phylogenetic relationship of GhABF2 homologs in plants. AT (*Arabidopsis thaliana)* OS (*Oryza sativa*), VV (*Vitis vinifera*), ST (*Solanum tuberosum*), POPTRO (*Populus trichocarpa*), RCOM (*Ricinus communis*), GM (*Glycine max*), MT (*Medicago truncatula*), CS (*Cucumis sativus*), CA (*Cicer arietinum*), CICLE (*Citrus clementine*), TC (*Theobroma cacao*), CM (*Cucumis melo*), EG (*Eucalyptus grandis*), POEUP (*Populus euphratica*), SI (*Sesamum indicum*), and JC (*Jatropha curcas*). The scale bar indicates 0.1 amino acid substitution per site. (**d**) Subcellular localization of GhABF2-GFP in rice protoplast. Scale bars = 10 μm.

The phylogenetic analysis of 28 unique Group A bZIP TFs members from *Arabidopsis* and rice revealed that they could be further divided into four distinct groups *i.e*. A-1, A-2 A-3, and A-4 (Fig. 1a). All members of Group A-1 and A-2 were involved in stress signaling, and many of them were found in ABA mediated expression pattern, indicated the conserved biological function of these TFs in plants. Further analysis revealed that Group A-1 and A-2 members share three conserved domain at their N-terminal (C1, C2, and C3) as well as a typical bZIP structure at their C terminal. Specifically, C3 and bZIP domains share very high similarity of their nucleotides and amino acids (Figs 1b and S1).

Based on the analysis of group A-1 and A-2 bZIP TFs characteristics, two pair of degenerate oligonucleotide primers were designed to amplify the possible members of cotton Group A-1 and A-2 bZIP TFs fragments (Table S1). DNA gel electrophoresis revealed that three PCR products with 660 bp, 590 bp, and 300 bp in size were amplified, respectively. Sequencing searches using the plant TFs database (http://planttfdb.cbi.pku.edu.cn/) revealed that they were similar to Group A-1 members of the *Arabidopsis* bZIP family, ABF2/AREB1, ABI5, and ABF3 protein, therefore, they were named GhABF2, GhABF3, and GhABI5, respectively (Fig. S2). Transcriptome data available at NCBI showed that the three genes were up-regulated by drought, high salinity, and exogenous ABA treatments in vegetative tissues, and the tissue-specific expression patterns and time-course expression profiling in different organs are similar under both control and stress conditions^42^ (Fig. S3a–c). Aforementioned finding demonstrated that ABF2/AREB1 acts as a key positive regulator of ABA-mediated stresses response in vegetative tissues of *Arabidopsis*. Thus, *GhABF2* was chosen for further analysis.

The 4,762 bp and 2,027 bp genome sequence and mRNA sequence were obtained by thermal asymmetric interlaced (TAIL)-PCR and rapid amplification of cDNA ends (RACE)-PCR, respectively (Fig. S4a,b). Sequence analysis showed that *GhABF2* contained 4 exons and 3 introns, and its 1,254 bp open reading frames (ORF) encoded a protein of 417 amino acids (Fig. S4a–c). GhABF2 shared 64% amino acid sequence identity with *Arabidopsis* ABF2/AREB1, which contains a typical bZIP domain at the C terminus. Databases and phylogenetic analysis revealed that GhABF2 protein also has 58% to 83% similarity with proteins of *Oryza sativa* (OS02G0766700), *Vitis vinifera* (NP_001268150), S*olanum tuberosum* (NP_001274925), *P. trichocarpa* (XP_002302435), *Ricinus communis* (XP_002510209), *Glycine max* (XP_003523938), *Medicago truncatula* (XP_003603049), *Cucumis sativus* (XP_004142865), *Cicer arietinum* (XP_004501562), *Citrus clementina* (XP_006434756), *Theobroma cacao* (XP_007017213), *Cucumis melo* (XP_008444613), *Eucalyptus grandis* (XP_010061749), *P. euphratica* (XP_011017194), *Sesamum indicum* (XP_011074426), and *Jatropha curcas* (XP_012071654) (Fig. 1c). Like *Arabidopsis* ABF2/AREB1, members of this group have a bZIP domain near the C terminus and related to the ABA-mediated signaling pathway for high stress tolerance (Figs S1 and S4c). Hence, GhABF2 may also function in ABA-mediated stresses response in cotton.

GhABF2 was predicted to be a nuclear protein by PredictNLS (https://rostlab.org/owiki/index.php/PredictNLS) and PSORT (http://www.genscript.com/psort/psort2.html) (Fig. S4c). To confirm the subcellular localization of GhABF2, a *35S::GhABF2*-*eGFP* vector containing eGFP-tagged *GhABF*2 was introduced into protoplasts. Confocal microscopic observation showed that the green fluorescent signals of GhABF2-eGFP was localized exclusively in nucleus, this confirmed that GhABF2 is a nuclear-localized protein and the predicted transit peptide was functional (Fig. 1d). Conclusively, these results indicated that GhABF2 was a typical bZIP TF and might be functional in response to stresses in cotton.

### Expression pattern of cotton *GhABF2*

**Figure 2:**
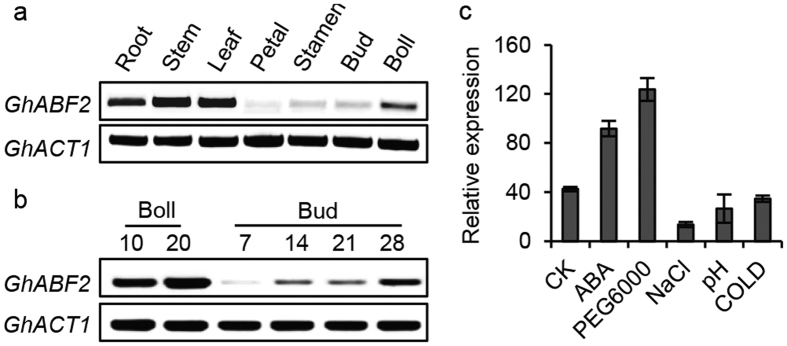
Analysis of *GhABF2* expression. (**a**) Expression of *GhABF2* in various organs, including root, stem, leaf, petal, stamen, bud and boll. (**b**) Expression of *GhABF2* in bud and boll at different developmental stages. (**c**) Effects of ABA and abiotic stresses on *GhABF2* expression. CK, control. **P ≤ 0.01; Student *t* test.

The temporal and spatial expression pattern of *GhABF2* by RT-PCR and quantitative RT-PCR (qRT-PCR) showed that *GhABF2* was preferentially expressed in root, stem and leaves. However, its expression was low but detectable in petals, stamen, bud, and boll (Fig. 2a). Moreover, the transcripts of *GhABF2* in bud and boll were gradually increased along with their development (Fig. 2b).

To investigate whether phytohormones and stress affect *GhABF2* transcription, we analyzed *GhABF2* expression in wide type seedling under the treatment of ABA and various stress. After 10-d incubation of ABA and stresses, the expression of *GhABF2* was induced significantly with ABA and high permeability (Fig. 2c). However, it was repressed by high-salinity, cold and high pH. These expression patterns indicated that *GhABF2* may function in multiply abiotic stress signal transduction.

### Expression of *GhABF2* in *Arabidopsis* showed enhanced drought and salinity tolerance

**Figure 3:**
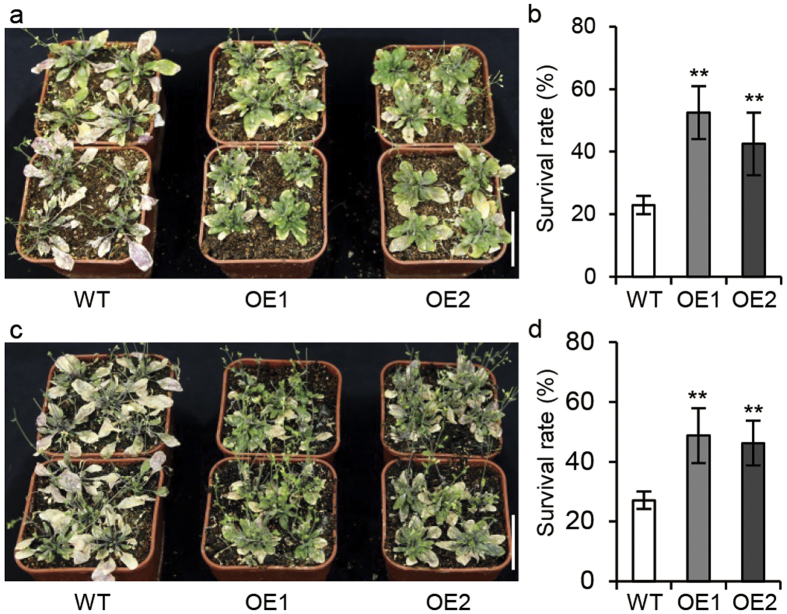
Overexpression of *GhABF2* enhanced drought and salt tolerance in *Arabidopsis*. (**a**) Phenotype of wild type and *Arabidopsis GhABF2* overexpression transgenic lines after 10 d drought treatments. Bar = 5 cm. (**b**) The survival rates of wild type and *Arabidopsis GhABF2* transgenic lines corresponding to (**a**). (**c**) Phenotype of wild type and *Arabidopsis GhABF2* overexpression transgenic lines after 7 d salt treatments with 200 mM NaCl. Bar = 5 cm. (**d**) The survival rates of wild type and *Arabidopsis GhABF2* transgenic lines corresponding to (**c**). WT, wild type. OE, overexpression transgenic lines. All experiments were repeated with three biological replicates (n = 48). **P ≤ 0.01; Student t test.

To evaluate the role of *GhABF2* during abiotic stresses, the *GhABF2* coding sequence was cloned into plant binary vector pBI121, and *GhABF2* overexpression *Arabidopsis* plants were developed though *Agrobacterium tumefaciens*-mediated transformation (Fig. S5). Transgenic seedlings were used for PEG6000 mediated osmotic stress and salt tolerance. In drought stress for 10 d, the survival rates of overexpression (OE) lines *i.e*. OE1 and OE2 was 52.5% and 42.5%, respectively. The wild type plants showed low survival rate 22.9% (Fig. 3a,b). Similarly, under salt stress for 7 d, the survival rates of OE1 and OE2 was 48.8% and 46.3%, respectively. However, the wild type showed 27.1% survival rate (Fig. 3c,d).

### Silencing of G*hABF2* by VIGS (virus-induced gene silencing) technology

**Figure 4:**
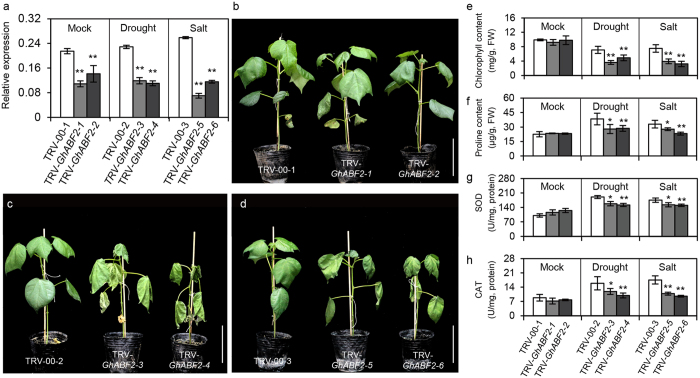
Silencing of *GhABF2* cottons showed sensitivity to drought and salt stress. (**a**) The expression of *GhABF2* in empty vector control (TRV-00) and *GhABF2*-silenced (TRV-*GhABF2*) cotton plants after three weeks infiltration under normal condition. Values are means ± SD of three replicates. *P ≤ 0.01, **P ≤ 0.01; Student *t* test. (**b**) Untreated control. Bar = 5 cm. (**c,d**) TRV-*GhABF2* cottons exhibited more sensitivity to drought (**c**) and salt (**d**) stress than wild plants. Bar = 5 cm. (**e–h**) Chlorophyll contents (**e**) proline contents (**f**) SOD activities (**g**) and CAT activities (**h**) of TRV-00 and TRV-*GhABF2* plants corresponding to (**b–d**). Values are means ± SD of five replicates. *P ≤ 0.01, **P ≤ 0.01; Student *t* test.

The tobacco rattle virus (TRV)-based VIGS construct was targeted specifically to the non-conserved region of *GhABF2* outside the bZIP domain to avoid interference with other bZIP proteins. Four week after *Agrobacterium tumefaciens* infiltration, the transcripts of *GhABF2* were significantly reduced in TRV-*GhABF2* plants compared with TRV-00 cotton, suggested that *GhABF2* was effectively silenced in cotton (Figs 4a and S6). After treated with drought or salt stress for three weeks, our TRV-*GhABF2* cotton observed more wilting and yellowing of leaves as compared with the control, which was consistent with the decline in chlorophyll contents (Fig. 4b–e). In addition, proline and activities of reactive oxygen species (ROS)-scavenging enzymes, such as superoxide dismutase (SOD) and catalase (CAT), were significantly lower in the TRV-*GhABF2* cotton leaves than in the TRV-00 plants (Fig. 4f–h).

### *GhABF2* enhanced the tolerance to drought and salinity stresses in cotton in the laboratory condition

**Figure 5:**
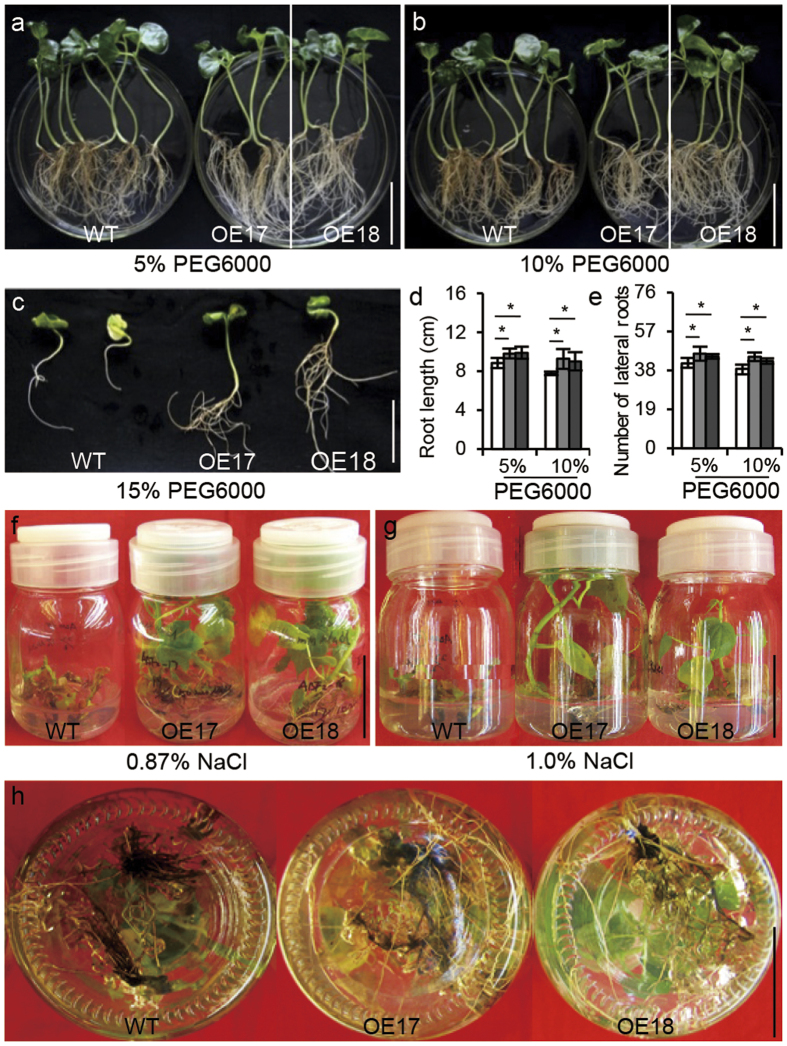
*GhABF2* significantly improved drought and salt tolerance of transgenic cotton under culture condition at vegetative stage. (**a–c**) Seedling vigor at 5% (**a**) 10% (**b**) and 15% (**c**) PEG6000 culture solution for 10 days, respectively. All experiments were repeated with three biological replicates (n = 5). (**d,e**) Root length (**d**) and number of lateral roots (**e**) corresponding to (**a,b**). *P ≤ 0.01, **P ≤ 0.01; Student *t* test. (**f,g**) Seedling vigor at 0.87% (**f**) and 1% (**g**) NaCl culture solution for 7 days, respectively. All experiments were repeated with three biological replicates (n = 5). (**f**) Root phenotypes corresponding to (**d**).

To further confirm the *GhABF2* function in response to stresses tolerance in cotton, we developed a number of transgenic cotton plants carrying a *CaMV 35S::GhABF2*. A set of 22 positive independent transgenic lines showed up-regulated expression of *GhABF2*. Among them, two transgenic lines OE17 and OE18 having 2.06- and 3.49-fold higher transcript level as compared to wild type respectively were chosen for further studies (Fig. S7a,b). Southern blotting analysis showed that the OE17 plants harboring a single copy of transgenic cassette, while OE18 plants containing double copy of integrated cassette (Fig. S7c). In addition, flanking sequence analysis showed that the OE17 insertion site was mapped in an inter-genic region of Chromosome A08-scaffold1925, from nucleotides 108,115 to 108,116. The OE18 insertion sites were mapped in scaffold 12678 and Chromosome D11 (from nucleotides 17,123,216 to 17,123,276), respectively (Fig. S7d,e). After incubation with 5%, 10%, and 15% PEG6000 for 10 days, these two independent transgenic lines, OE17 and OE18, showed enhanced drought tolerance. The leaves maintained their chlorophyll pigments for a longer period. The progression of leaf yellowing and vitrification occurred at a much slower rate (Fig. 5a–c). The transgenic lines *i.e*. OE17 and OE18 had well developed root system with longer primary roots (Fig. 5d), more lateral roots number (Fig. 5e), larger total root surface area (Fig. S8a), and longer total root length as compared to wild type (Fig. S8b).

Emergence and seedling stage of cotton are more sensitive to salinity than other growth stages. Two week old seedling of two transgenic lines OE17 and OE18 showed significant tolerance, having higher plant height and larger leaves at 0.87% and 1% NaCl treatment for two weeks (Fig. 5f,g). The leaves of the wild type seedling turned yellow and more necrosis because of salt toxicity, whereas *GhABF2* overexpression seedlings were still green. The *GhABF2* overexpression cotton seedling also had well-developed root system with larger total root surface area (Fig. S8c), longer total root length (Fig. S8d), and low necrosis in contrary to their wild type (Fig. 5h).

### *GhABF2* overexpression lines improved cotton tolerance to drought and salinity in greenhouse

**Figure 6:**
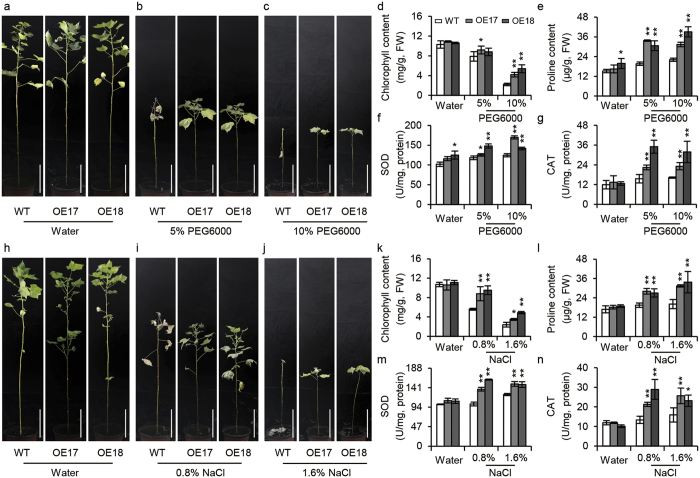
*GhABF2*-overexpressing transgenic cotton significantly enhances stress tolerance in greenhouse. (**a–c**) Phenotypes of wild type and transgenic plants after six weeks treatment with water (**a**) 5% (**b**) and 10% (**c**) PEG6000. All experiments were repeated with three biological replicates. Scale bar = 6 cm. (**d–g**) Chlorophyll contents (**d**) proline contents (**e**) SOD activities (**f**) and CAT activities (**g**) of transgenic and wild-type plants corresponding to (**a–c**). Values are means ± SD of three replicates. *P ≤ 0.01, **P ≤ 0.01; Student *t* test. (**h–j**) Phenotypes of wild type and transgenic plants after six weeks treatment with water (**h**) 0.8% (**i**) and 1.6% (**j**) NaCl. All experiments were repeated with three biological replicates. Scale bar = 6 cm. (**k–m**) Chlorophyll contents (**k**) proline contents (**l**) SOD activities (**m**) and CAT activities (**n**) of transgenic and wild-type plants corresponding to (**h–j**). Values are means ± SD of five replicates. *P ≤ 0.01, **P ≤ 0.01; Student *t* test.

We further evaluated the drought and salt tolerance of the *GhABF2*-overexpressing cotton lines in the greenhouse. Two sets of 30-d old transgenic lines OE17 and OE18 seedlings were continuously watered with 5% PEG6000, 10% PEG6000, 0.8% NaCl, and 1.6% NaCl, respectively for six weeks. After treated with PEG6000 and salt we observed as significant increase in drought and salt tolerance as transgenic plants showed delay in senescence with more chlorophyll as compared to control (Fig. 6a–d,h–k). During the 5% and 10% PEG6000 treatment, proline and activities of reactive oxygen species (ROS)-scavenging enzymes, such as SOD and CAT, were increased in both the wild type and transgenic lines, however, *GhABF2*-overexpressing cotton exhibited more significant increases compared in the wild-type plants (Fig. 6e–g). Similar kinds of results were also observed under 0.8% and 1.6% salt treatment (Fig. 6l–n). These results were inconsistent with phenotype of *GhABF2* transgenic plants having decreased rate of chlorophyll degradation.

### Transcriptome analysis of the GhABF2

**Figure 7:**
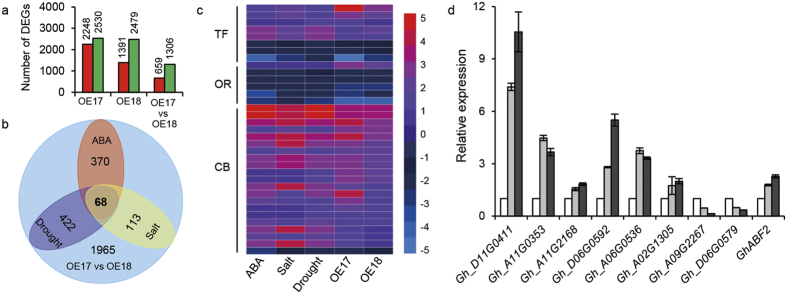
RNA-Sequencing analysis of *GhABF2*-overexpressing cotton leaves transcriptome. (**a**) Changes in gene expression profile between control, OE17, and OE18. DEGs, differently expressed genes. vs, versus. The red bar represents up-regulated gene, and the green bar represents down-regulated gene. (**b**) Venn diagram of DEGs in cotton seedling leaves between OE17 vs OE18 and different abiotic stress conditions. The DEGs data response to ABA, drought, and salt treatment were extracted from GEO at NCBI (accession number GSE50770). (**c**) Heatmap of the part of 68 DEGs. TF, transcription factor. OR, oxidation reduction. CB, chlorophyll biosynthetic. (**d**) Expression of TF genes by qRT-PCR. Values are means ± SD of three replicates. *P ≤ 0.01, **P ≤ 0.01; Student *t* test.

We performed RNA-sequencing analysis using WT, OE17, and OE18 transgenic cotton lines and a comparison was done with the global transcriptional profiles using the age-matched leaves. Among the 1965 differential expressed genes (DEGs) with alteration of 2-fold or more, 659 were up-regulated and 1306 were down-regulated in both OE17 and OE18 lines (Fig. 7a, Table S2). Enrichment analysis of DEGs identified ‘plant hormone signal transduction’ as a top annotated pathway (Fig. S9a,b). For comparative analysis, publicly available transcriptomic sequence data were mined. Notably, 370 (18.8%), 113 (5.8%), and 422 (21.5%) of the OE17 vs OE18 DEGs overlapped with those of the ABA, salt and drought-treated cotton seedling respectively. A total of 68 DEGs were up- or down-regulated in OE17 and OE18 under ABA, drought and salt treatment (Fig. 7b and Table S2), including eight transcription factor (TF) genes, six oxidation reduction (OR) process related genes, and 31 chlorophyll biosynthesis (CB) associated genes (Fig. 7c). For upregulated genes, obvious over-representation was observed for thiamine biosynthetic, response to stress, and oxidation reduction in the biological process category (Table S2). Recently work revealed that thiamine biosynthetic genes were closely related to stress response in plant^43^,^44^,^45^. Consistently, genes encoding iron-sulfur cluster binding, nucleic acid binding, and oxidoreductase activity were significantly over-presented in the molecular function category. For downregulated ones, genes encoding protein binding were clearly over-represented in molecular function category (Table S2). Moreover, we verified the different expression of the DEGs in OE17and OE18 plants by qRT-PCR (Figs 7d and S10b,c). Further, the expression levels of up-regulated DEGs in *GhABF2*-overexpression plants were significantly down-regulated in *GhABF2*-silenced cotton, whereas suppressed DEGs were activated (Fig. S11). These results strongly demonstrated that *GhABF2* modulated specific ABA responses to enhance drought and salt tolerance in cotton.

### Overexpression of *GhABF2* leads to increased cotton yield under stress condition

**Figure 8:**
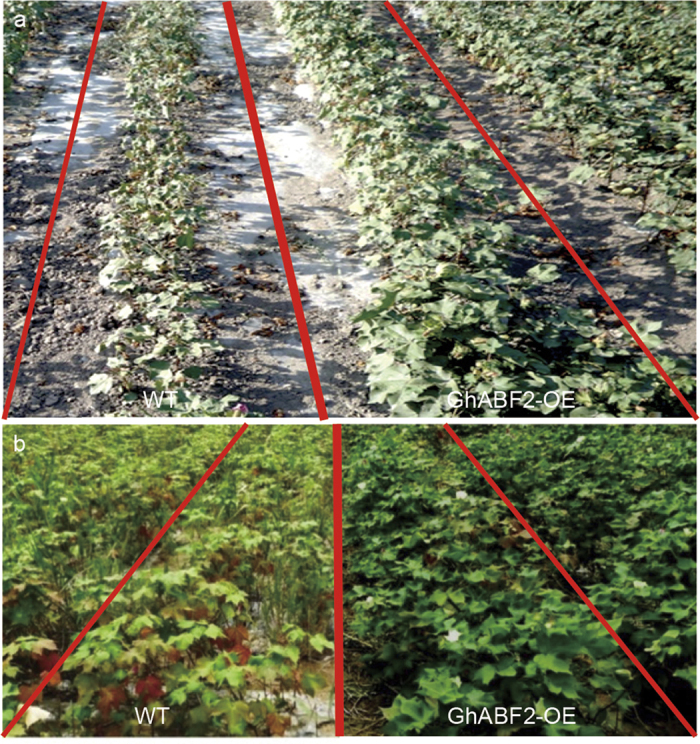
Field evaluation of the *GhABF2-OE* transgenic cotton. (**a**) Field trait of the *GhABF2* overexpression transgenic cotton in Shihezi, Xinjiang Province, northwest of China. Pictures were taken after 60 days treatment under natural drought conditions. (**b**) A field view showing the phenotypes of *GhBAF2* overexpression and control lines in salt contamination field in Dongying, Shandong Province, east of China. The pictures were taken 180 days after sowing.

The field performance of transgenic cotton line OE17 and OE18 apparently did not showed any significant differences with wild type cotton under normal conditions. The *GhABF2* overexpression transgenic lines *i.e*. OE17 and OE18 showed significantly tolerance to drought stress as compared to wild type in the fields in Shihezi, Xinjiang province, northwest of China, where rainfall is extremely scarce during the entire growing season (Fig. 8a). Statistical analysis showed that the number of fruit branches per plant of two lines, OE17 and OE18, increased to about 3.75 and 3.74, respectively, compared with wide type (2.60). The number of bolls per plant of OE17 and OE18 increased to 4.33 and 4.31, respectively, compared to wide type (2.88). This improvement led to an increase in cotton fiber yield of about 46.0% and 13.8% in both lines OE17 and OE18, respectively (Table 1). Thus, drought tolerance of *GhABF*2 overexpression lines in the field were further confirmed in a trial conducted on a test plot.

We also evaluated the salinity tolerance of *GhABF2* overexpression cottons lines in the salt contamination field in the Dongying, China’s eastern coastal city of Shandong province. Similarly to drought stress response, the *GhABF*2 overexpression transgenic cotton showed not only enhanced salt tolerance, having better agronomic traits in saline-alkali field (Fig. 8b). The data depicted that transgenic cotton lines produced higher plant height, more number of fruiting branches, number of bolls, and fiber yield contrary to wild type (Table 1), suggesting that upregulated *GhABF2* transcripts in cotton increased salt tolerance and improved fiber production under high salt field.

## Discussion

Plants being sessile organisms do not have tendency to move away to avoid the adverse environmental effects and ultimately they have to face diverse environmental conditions. The adoption of technologies to enhance the crop production under biotic and abiotic stresses is imperative. Therefore, engineering salt or drought tolerance is an objective of plant breeder and has potential to develop novel salt or drought tolerant germplasm. The availability of draft genome sequence of cotton has facilitated the research work on functional genomics^42^,^46^,^47^,^48^ and research on identification, characterization, and transformation of stress responsive genes has been done by many geneticists^2^,^25^,^35^,^37^,^39^. A larger number of genes are found to be associated with stress tolerance in plants, but the contribution of each gene is little towards deploying the overall stress tolerance^49^. The genetic engineering of plant using transcription factors (TFs) could be a yielding strategy for obtaining stress tolerant plant because of their involvement in regulating the expression of several genes related to biotic and abiotic stresses^3^. Therefore, we cloned, characterized and transformed a novel endogenous gene *GhABF2* encoded for bZIP TF to deploy the resistance against drought and salinity.

*GhABF2*, a bZIP Group A family gene, is involved in ABA-regulated drought and salinity stress response signaling pathway. First, *GhABF2* overexpression transgenic lines displayed a significant enhanced drought and salt tolerance both in *Arabidopsis* and cotton, while *GhABF2*-silenced cottons were much more sensitive to abiotic stress. Second, genome-wide expression profiling by RNA sequencing reveals that *GhABF2* improves drought and salt tolerance by regulating at least 68 genes simultaneously associated with ABA, drought, and salt stress responses. Third, the *GhABF2*-overexpressing cottons were better protected from oxidative by increasing the levels of proline and two activates of reactive oxygen species-scavenging enzymes, SOD and CAT. Fourth, the transgenic lines with activation of *GhABF2* had no impact on most of agronomic traits under normal condition, but they had better agronomic performance and ultimately led to higher fiber yields under drought and salt conditions in the field. Those results clearly demonstrate that GhABF2 TF acts as an important regulator for enhancing the tolerance against drought and salinity, and could be a potential source for breeding drought and salinity tolerant cotton genotypes.

Numerous studies, both genetics and biochemical, revealed that ABA acts as a key mediator in enhancing plant multiple stress tolerance after it was identified in the 1960s^50^. During abiotic stress, a significant increase in endogenous ABA levels was detected in many plants, concomitantly with up- or down- regulation of a series of ABA signaling genes and stress-related genes^1^. Furthermore, overexpression of ABA biosynthesis genes, such as *NCED*^51^, *ZEP*^52^, and *LOS5*^53^, led to increase ABA production and enhance stress tolerance in transgenic plants. Prior work has identified that Group A of bZIP TFs regulate ABA-dependent stress responses by inducing stresses-associated genes expression^17^. Sequence comparison indicated that GhABF2 have significant similarity to others factors in bZIP domain. Notably, in our RNA-Sequencing data, a large proportion of DEGs were determined to responsible to ABA. Moreover, 68 DEGs were significantly up- or downregulated similarly under drought and salt treatment. Based on these results, we can reasonably conclude that GhABF2 control abiotic stress response by mediating ABA signaling pathway. Interestingly, the expression of *GhABF2* was induced by PEG6000 but repressed by salt, constitutive overexpression of *GhABF2* significantly enhanced plants tolerance to drought and salt. These expression patterns were consisting with the expression profiling reported by Zhang *et al*. (http://www.ncbi.nlm.nih.gov/bioproject/PRJNA248163/)^42^. It was reported that three bZIP TFs *OsbZIP71*^3^, *OsbZIP52*^54^, and *ZAT10*^55^, act as both positive and negative regulator in abiotic/biotic stress in plants. Thus, our data infer that there might be different regulation mechanism of *GhABF2*-mediated stress response for drought and salinity.

Furihata *et al*. (2006) reported that ABA-dependent phosphorylation of AREB/ABF-type transcription factors by SnRK2 protein kinase may be involved in the activation of AREB/ABFs in *Arabidopsis*. Our sequence analysis also revealed that GhABF2 also contains two typical target sequences for Ser/Thr protein kinases, R-X-X-S/T (putative targets for CDPK) and S/T-X-X-E/D (putative targets for CK II) in three conserved regions C1, C2, and C3 domains (Fig. S12). These results suggested that like *Arabidopsis* there might be ABA-dependent multisite phosphorylation of GhABF2 which regulates its own activation in cotton. Because of the higher similarity between the sequence and structure, the members of Group A-1 and A-2 bZIP TFs are likely to regulate many of the same target genes, thereby they are possibly functional redundancy homologs in *Arabidopsis*^22^,^56^. In our studies, three cotton Group A TFs, GhABF2, GhABF3, and GhABI5 are in the same clade in the phylogenetic tree of the bZIP subfamily (Fig. S2). The tissue-specific expression profiling and induced expression patterns in different tissues are similar under both control and stress conditions (Fig. S3a–c). In addition, target sequences for Ser/Thr protein kinases in three conserved regions proved to be coincident in GhABF2, GhABF3 and GhABI5 (Fig. S12). These results implying the potential functional similarity of GhABF2, GhABF3 and GhABI5 as mediator of ABA-signaling and stress response processes in cotton. However, they also show several different expression patterns under different treatment and developmental processes (Fig. S3a–c). This discrepancy in the three genes may be attributed to different mechanism in regulating developmental processes and specific abiotic stresses response in different tissues. To this end, a detail analysis of these genes expression pattern and transgenic lines phenotype are imperative to be investigated in the future.

Intracellular ROS was accumulated under diverse environmental stimuli, such as drought, salinity, cold, and heat stress^25^,^34^, causing damages in lipid member and biomolecules, and trigger the expression of several kinds of genes, including TFs, programmed cell death, leaf senescence, and chlorophyll degradation^57^,^58^. Two antioxidant enzymes SOD and CAT are considered as major scavenger of ROS in all aerobic organisms, and theirs activity are tightly linked with lower rates of oxidative damage and cell death under abiotic stress^59^,^60^. In our study, SOD and CAT activities were much higher in the *GhABF2*-overexpressing cottons, while lower in the *GhABF2*-silenced lines, indicating that GhABF2 TF protecting the cottons from oxidative damage partly dependent on promoting ROS-scavenging capability. Accumulation of free proline, an important osmolytes in cells, is thought to protect plants during abiotic stress^25^,^61^. The significant reduced proline levels detected in the TRV-*GhABF2* cottons correlated well with the drought- and salt-sensitive phenotype under both PEG6000 and salt treatment. Therefore, the accumulation of free proline in *GhABF2*-overexpressing plants may be another important factor to enhance abiotic tolerance.

Cotton, being a perennial plant with indeterminate growth habit has complicated response mechanism to drought and salinity. These responses to abiotic stresses are not linear but comprises of network evolved to deal with changing environmental conditions and plant immobility. ABA is stress hormones that mediate different process in growth and development, gene expression and response to environmental stresses^62^. In our study overexpression of *GhABF2* encoded for TF bZIP which is mainly related to ABA mediated stress response ultimately led to significant increase in yield under drought and salinity condition. This might be due to the fact that drought/salinity shares the same protective mechanisms because both are involved in dehydration stress and ABA possess hydrostatic effects on plants, apart from this enhanced sink strength *i.e*. carbohydrates has been reported in many plant species^63^,^64^. In addition to this both stresses can limit the process of photosynthesis, respiration, flowering, bolls and ion uptake in cotton, resulting in yield losses^65^,^66^,^67^. Previously Mittal *et al*.^62^ reported that transgenic lines for drought tolerance have more plant height with more leaf and total biomass. We also observed a significant increase in plant height under drought and salinity in transgenic lines as compared to their wild type. These finding suggested the positive role of *GhABF2* toward the increase of plant height by mitigating the stress condition.

Taken together, overexpression of *GhABF2* in transgenic *Arabidopsis* and cotton enhanced drought and salt tolerance. The transgenic plants improved multiple characteristics related to abiotic stress, including well-develop root system, increased proline, SOD and CAT levels, reduced chlorophyll degradation, and delayed leaf cell death in cotton. More importantly, the transgenic cotton showed encouraging results in enhancing drought and salt stress both in laboratory and field experiment, and leads to remarkable higher cotton fiber yields under stress condition. Thus, identification and characterization of *GhABF2* could facilitate the rational applications in actual agriculture practice.

## Material and Methods

### Plant materials and growth conditions

The sterilized cotton seeds of cultivars Sumian-12, a cultivated variety in Jiangsu and Shandong Province, China, were germinated on half strength MS solid medium (Murashige and Skoog medium) for 7 d maintaining 12-hour-light (28 °C)/12-hour-dark (25 °C) photoperiod, *GhABF2* induction analysis was performed by transplanting the cotton seedling to the corresponding medium have 50 μM ABA, 200 mM PEG6000, 200 mM NaCl, pH = 11 (high pH, hereinafter referred to “pH stress”), and 4 °C for 2 days. Five plants were collected for RNA isolation. For drought tolerance test, 2-week-old seedlings were transplanted to the half strength MS media having 5%, 10%, and 15% PEG6000 for 10 d. To examine the possible role of *GhABF2* during salt stress in cotton, 2-week-old seedlings were transplanted to half strength media containing 0.87% and 1%NaCl and seedling were incubated for 7 d. All experiments were repeated five times independently.

*Arabidopsis thaliana* seeds of Col-0 and *GhABF2-OE* transgenic plants were sown on Petri dishes containing MS salts with 0.8% (w/v) phytoagar. After vernalization at 4 °C for 2 d, the dishes were moved to a growth chamber at 22 °C with 70% humidity. After 12 d of germination, seedlings were transplanted to pots containing a peat soil:vermiculite:perlite mixture (3:9:0.5, v/v/v).

### Cloning of the *GhABF2*

With reference to the structural features of bZIP Group A-1 and A-2 TFs in *Arabidopsis* and rice, four degenerate primers were designed to amplify cotton Group A bZIP TFs fragments. The Genome sequence of *GhABF2* was isolated by Genome Walker PCR (TaKaRa, Dalian, China). Full-length open reading fragment (ORF) of *GhABF2* was isolated by Invitrogen RACE system. The primers used for PCR are listed in Table S1.

### RNA extraction, cDNA preparation, and gene expression analysis

Total RNAs were extracted based on the method described by Zhou *et al*.^68^. RNA was reverse transcribed using the ReverTra Ace qPCR RT Master Kit (Toyobo, Japan). Quantitative real-time (qRT)-PCR was performed with Chromo 4 real-time PCR detection system following manufacturer’s instructions (Bio-Rad, CFX96). The data were analyzed with Opticon monitor software (Bio-Rad). Cotton *GhACT7* was used as an internal control. The primers used for qRT-PCR are listed in Table S1. Student’s *t*-test was used for statistical analysis.

### Subcellular Localization

To determine localization of GhABF2 protein in plant cells, full-length *GhABF2* coding sequence was amplified using primers listed in Table S1 and inserted into *CaMV 35S::GFP* vector. The binary vector *CaMV 35S::GhABF2-GFP* was subsequently transformed into rice protoplasts using the polyethylene glycol method. After overnight incubation in the darkness, the protoplasts expressing eGFP were imaged by a confocal laser scanning microscope (LSM510, Zeiss, Germany). Composite figures were prepared using Zeiss LSM Image Browser software.

### Sequence and phylogenetic analysis

Nucleotide and amino acid sequences were analyzed using DNAstar (DNAstar, Masidon) and DNAMAN analysis program (Lynnon Biosoft, USA). GhABF2 homologs were detected by BLASTp using the entire amino acid sequence of GhABF2 as a query in the National Center for Biotechnology Information Website (http://www.ncbi.nlm.nih.gov/). Multiple alignments of the homologs were performed by ClustalX version 2.0 with the default parameters and manually adjusted. The neighbor-joining method of the MEGA 4.1 software was used to test the confidence of topology.

### Plasmid construction and plant transformation

To construct the pBI121-*GhABF2* Plasmid, the entire coding region of *GhABF2* was amplified by PCR with *Pst* І-*Xho* І linker primers and cloned into the *Pst*І and *Xho*І sites of pBI121. Primers used for vector construction are listed in Table S1. *Agrobacterium tumefaciens* (LBA4404) mediated transformation was done by vacuum infiltration method and *GhABF2* overexpression transgenic *Arabidopsis* were develop using vectors *pBI121-GhABF2*. *GhABF2* overexpression transgenic cotton was developed using the hypocotyl segments of upland cotton cultivar, Sumian-12. After regeneration, the plantlets were transferred to pots for further growth and to have T0 seeds.

### Southern hybridization analysis

Total genomic DNA (40–60 μg) were extracted from fresh cotton leaves, and southern hybridization was carried out according to previously-reported methods^69^. The probe for *GhABF2* was the 782 bp PCR fragments amplified with the primers listed in Table S1.

### Drought and salt tolerance assay in *Arabidopsis*

The drought and salt tolerance assay for *Arabidopsis* seedlings was performed using wild type and *GhABF2* OE *Arabidopsis* plants grown under 16 h illumination of 50 ± 10 μM photons m^−2^s^−1^ at 22 °C and 35 ± 5% relative humidity. Drought stress was imposed by withholding the water for 10 d. The salinity tolerance assay was performed using 7 d old wild type and *GhABF2* OE *Arabidopsis* lines. Both lines were transferred to half-strength MS medium with 200 mM NaCl for saline stress.

### Drought and salt tolerance assay in cotton

The 7 d old cotton seedlings of wild-type and *GhABF2*-OE were transferred to solid half-strength MS medium, half-strength MS medium infused with 5%, 10%, and 15% PEG6000 for drought stress, and half-strength MS medium with 0.87% and 1% NaCl for salt stress.

To evaluate drought and salt tolerance of *GhABF2* transgenic cotton in Greenhouse, the homozygous transgenic cotton OE 17 and OE18 and wild-type plants were separately germinated in well-watered soil in the Greenhouse at Chinese Academy of Agricultural Sciences, Beijing. For drought treatment, a set of 30-d old cotton seedlings were continually watered with 5% and 10% PEG6000 and for salt treatment 30-d old seedlings were continually watered with 0.8% and 1.6% salt for six weeks. The root morphological parameters were measured as described by Qu *et al*.^70^.

### Chlorophyll measurement

Chlorophyll was extracted from 50 mg leaf tissue (fresh weight) and determined by measuring the absorbance at 652 nm using a Tecan Infinite M200 multimode reader (Tecan Group Ltd) as described previously^71^.

### Measurement of proline content

Leaves of similar developmental stages from stress-related lines and control plants were used for proline contents measurement. Proline was assayed as described previously^25^.

### Determination of SOD and CAT activity

The measurement of SOD and CAT activity was performed according to a previously described method^25^. Total protein content was measured using the Bradford protein assay kit (Sangon Biotech, Shanghai, China).

### RNA sequencing and data analysis

RNAs extracted from 6-week old leaves of wide type and *GhABF2*-overexpressing transgenic plants were used for the RNA sequencing. For direct comparison, three libraries, WT, OE17, and OE18 were prepared in the same manner and run side by side by REALGEN Company on Illumina Hiseq TM 2000 platform. After removal of adaptor sequences, duplication sequences, ambiguous reads and low-quality reads, 11,695,933 (WT), 12,148,169 (OE17), and 11,633,121 (OE18) high-quality clean reads were generated. About 84.3%, 85.3%, and 85.6% reads of WT, OE17 and OE18 were mapped uniquely to the land cotton genome respectively through the Bowtie software. Differentially expressed genes (DEGs) were analyzed by the Cufflinks software with the FPKM (fragments per kilo bases per million reads) measurement. Genes with more that 2.0-fold change and P values ≤ 0.05 in both OE17 and OE18 libraries were regarded as DEGs. The DEGs data response to ABA-, drought, and salt treatment were download from GEO (Gene Expression Omnibus) at the National Center for Biotechnology Information (NCBI, http://www.ncbi.nlm.nih.gov/geo/) with the accession number GSE50770^35^. Functional annotation to the DEGs was analyzed through the website of MASCOTTON (http://mascotton.njau.edu.cn/). The percentage refers to the ratio of genes relative to the total up-regulated or down-regulated DEGs in each functional category. The DEGs with log2 ratios ≥ 1.00 or ≤ −1.00 (only GO Slim IDs with P values ≤ 0.05) were analyzed. The RNA-SEQ data were deposited at SRA at the National Center for Biotechnology Information (NCBI, http://www.ncbi.nlm.nih.gov/sra/) with the accession number SRP078316.

### VIGS in cotton followed by drought and salt treatment

The TRV and positive control TRV-*CLA1* (*G.barbadense cloroplastos alterados 1*) vector and *Agrobacterium tumefaciens* for VIGS were performed according to a previously described method^72^. Fragment to construct TRV-*GhABF2* was amplified from the cDNA of *Gossypium hirsutum* cvY18R. PCR fragment was digested with *Bam* HІ and *Kpn* І and then ligated into the TRV-00 plasmid. TRV vectors were agro-infiltrated as described into the cotyledons of 10-day-old seedling of Y18R. After three-week infiltration, TRV-00 and TRV-*GhABF2* cottons were used for drought and salt treatment.

### Agronomic traits under drought and salt stress field

The ultimate aim of drought and salinity tolerance research is to improve the ability of plants to maintain their growth and yield under stress relative to non-stress environment in the field conditions. Two independent T0 transgenic plants were grown to maturity in plots. Self-fertilized seeds (T1) were harvested and sown in the experimental field. T2 seeds harvested from positive T1 lines and transgenic homozygous plants were selected based on the results of PCR detection of kanamycin resistant gene. The transgenic homozygous lines were selected and self-fertilized to obtain T3 progeny. Such selections were continued to T5 progeny until the agronomic traits of the transgenic homozygous lines were stabilized. The performance of T5 homozygous *GhABF2* overexpression transgenic and its wild-type cotton lines was evaluated under normal, salt and drought prone areas during normal growing season of cotton. The field trait under the normal environmental conditions was conducted at experimental field of Chinese Academy of Agriculture Sciences, Beijing.

### Drought and salt stress in field

The drought tolerance of both transgenic and wild type cotton was assessed at Shihezi, Xinjiang province, northwest of China, where rainfall is extremely scarce during the entire growing season. One month after germination when plants were established, the irrigations were stop to develop the drought stress. Similarly, salt tolerance of *GhABF2* overexpression transgenic lines and wild type lines were evaluated in salt contamination field (salt concentration ≥ 0.3%) at Dongying, China’s eastern coastal city of Shandong province.

### Data recording for morphological traits

In all field traits, plants were grown following a randomized complete block design having three replications. Each tested transgenic cotton lines was planted in about 22.5 m^2^ experimental plot with three repeats (600 plants for each). The plant to plant distance was 12.5 cm and row to row spacing was 30 cm. All recommended plants protection measures were adopted from sowing to the harvesting. Fifty plants from each repeat were randomly selected for important agronomic traits including plant height, number of fruit branches per plant, and number of bolls per plant were measure on a single-plant basis. Plant height was determined as the height of the main stem at the boll opening stage. Vegetative shoot and fruiting branch of the main stem were separated manually for measurements of number of fruit branches per plant. All available cotton bolls from a single plant were collected for measurements of number of bolls per plant. All natural-opened bolls from a single plot were collected and dried at 37 °C in an oven, and 100 randomly picked bolls were used for boll weight measurements. All of the opened bolls before and after frost in a single plot were collected and treated as described above for measurement of actual cotton yield.

## Additional Information

**How to cite this article**: Liang, C. *et al*. GhABF2, a bZIP transcription factor, confers drought and salinity tolerance in cotton (*Gossypium hirsutum* L.). *Sci. Rep*. **6**, 35040; doi: 10.1038/srep35040 (2016).

## Supplementary Material

Supplementary Information

Supplementary Data S1

## Figures and Tables

**Table 1:** Agronomic trait and cotton fiber yields of transgenic lines in the field under natural drought conditions (Shihezi, Xinjiang Province) and saline-alkali field (Dongying, Shandong Province).

Genotype	Plant height (cm)	Number of fruit branches (per plant)	Number of bolls (per plant)	Boll weight (g)	Cotton yield (kg/plot)
Drought					
WT	85.33 ± 9.00	2.60 ± 0.40	2.88 ± 0.97	1.98 ± 0.88	2.42 ± 0.14
OE17	93.00 ± 14.61^*^	3.75 ± 0.33^*^	4.33 ± 0.48^*^	2.18 ± 0.48	3.64 ± 0.81^*^
OE18	88.33 ± 8.18^*^	3.74 ± 0.25^*^	4.31 ± 0.17^*^	2.13 ± 0.34	3.62 ± 0.40^*^
Salt					
WT	78.20 ± 11.27	10.00 ± 1.70	9.00 ± 3.17	2.49 ± 0.32	5.69 ± 0.50
OE17	89.65 ± 9.72^*^	12.20 ± 1.92^*^	10.70 ± 3.77^*^	2.28 ± 0.27	6.64 ± 0.07^*^
OE18	79.85 ± 7.93^*^	11.85 ± 1.62^*^	10.50 ± 4.57^*^	2.10 ± 0.18	6.80 ± 0.48^*^

Values are means ± SD (*P < 0.05). WT, wide type.
