# Supplementary material for: GhABF2, a bZIP transcription factor, confers drought and salinity tolerance in cotton (Gossypium hirsutum L.)
Source: Sci Rep. 2016 Oct 7;6:35040. doi: 10.1038/srep35040 (PMC5054369; doi:10.1038/srep35040)
Supplement: Supplementary Information [file srep35040-s1.pdf]

Supplementary Information

GhABF2, a bZIP transcription factor, confers drought and salinity tolerance in cotton (*Gossypium hirsutum* L.)

Chengzhen Liang[1], Zhaohong Meng[1], Zhigang Meng[1], Waqas Malik [1, 2], Rong Yan[1, 3], Khin Myat Lwin[1,4], Fazhuang Lin[1], Yuan Wang[1], Guoqing Sun[1], Tao Zhou[1],Tao Zhu[1], Jianying Li[5], Shuangxia Jin[5], Sandui Guo[1*] and Rui Zhang[1*]

[1]Biotechnology Research Institute, Chinese Academy of Agricultural Sciences, Beijing 100081, China

[2]Department of Plant Breeding and Genetics, Bahauddin Zakariya University, Multan, Pakistan

[3]College of Agronomy and Biotechnology, Southwest University, Chongqing 400715, China

[4]Biotechnology Research Department, Ministry of Science and Technology, Naypyidaw, Myanmar

[5]National Key Laboratory of Crop Genetic Improvement, Huazhong Agricultural University, Wuhan, Hubei 430070, China

[*]Correspondence address:
Rui Zhang and Sandui Guo

Biotechnology Research Institute, Chinese Academy of Agricultural Sciences, National Key Facility for Crop Gene Resources and Genetic Improvement, Beijing 100081, China

E-mail: zhangrui@caas.cn or guosandui@caas.cn
Tel & fax: +86-10-82106127

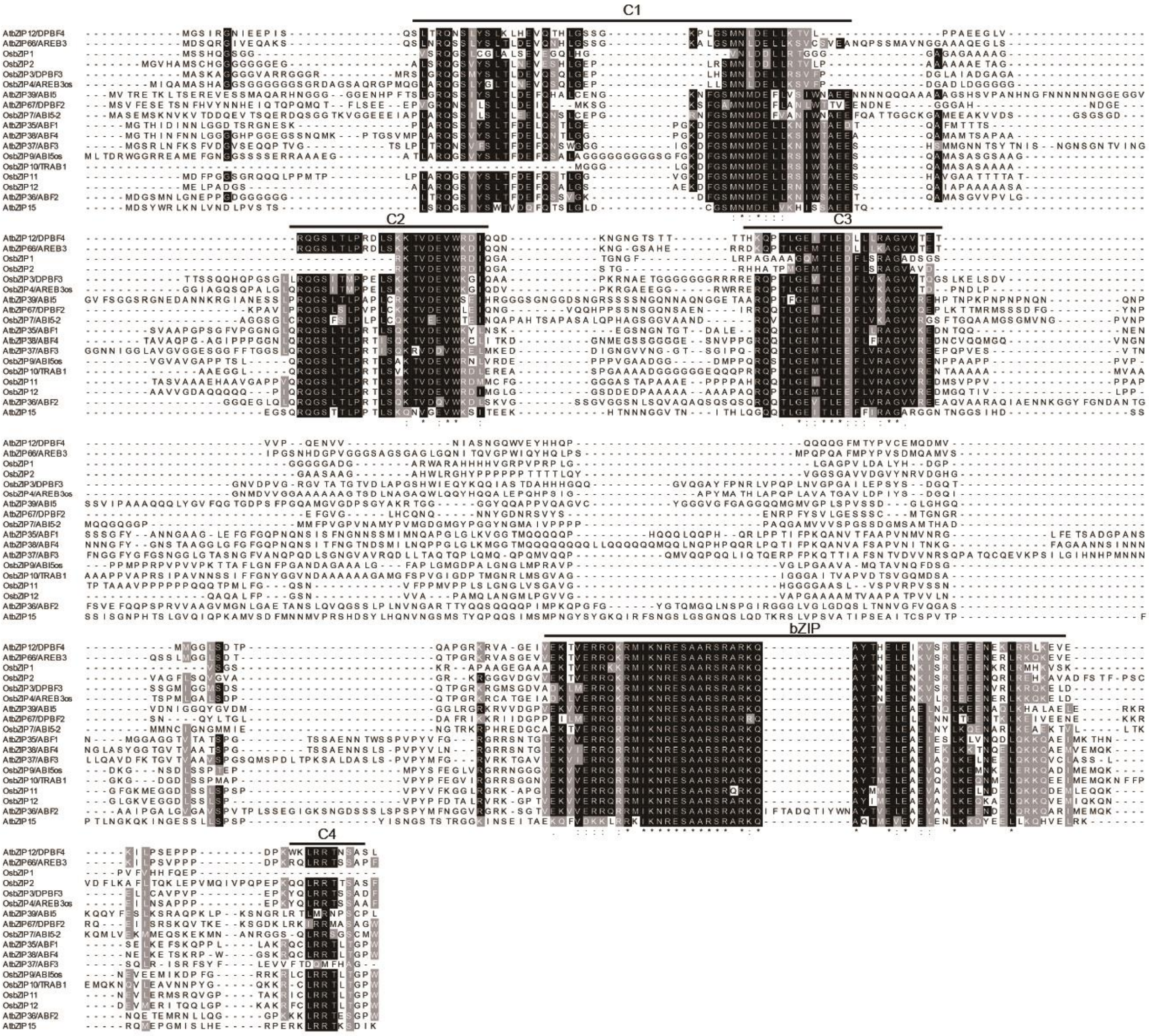

Figure S1. Protein sequence alignment of Group A bZIP transcription factors in *Arabidopsis* and rice using Cluster X. A conserved bZIP domain specific to bZIP proteins are indicated at C-terminus, while four conserved motifs (C1, C2, and C3) at N-terminal and C4 at C-terminal specific to Group A are indicated. Conserved amino acids are highlighted in shades of black and gray. White letter with black background (100% identity), white letter with gray background (60%).

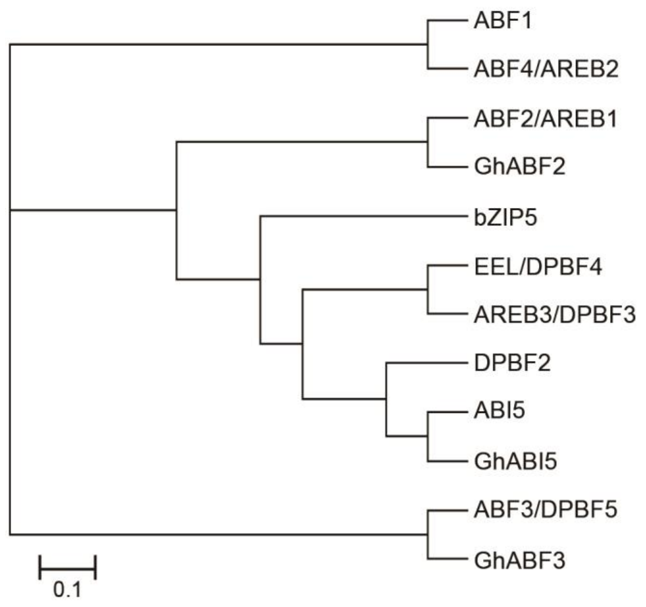

Figure S2. Phylogenetic relationship of GhABF2, GhABF3, and GhABI5 homologs in Group A bZIP TFs in *Arabidopsis*. The scale bar indicates 0.1 amino acid substitution per site.

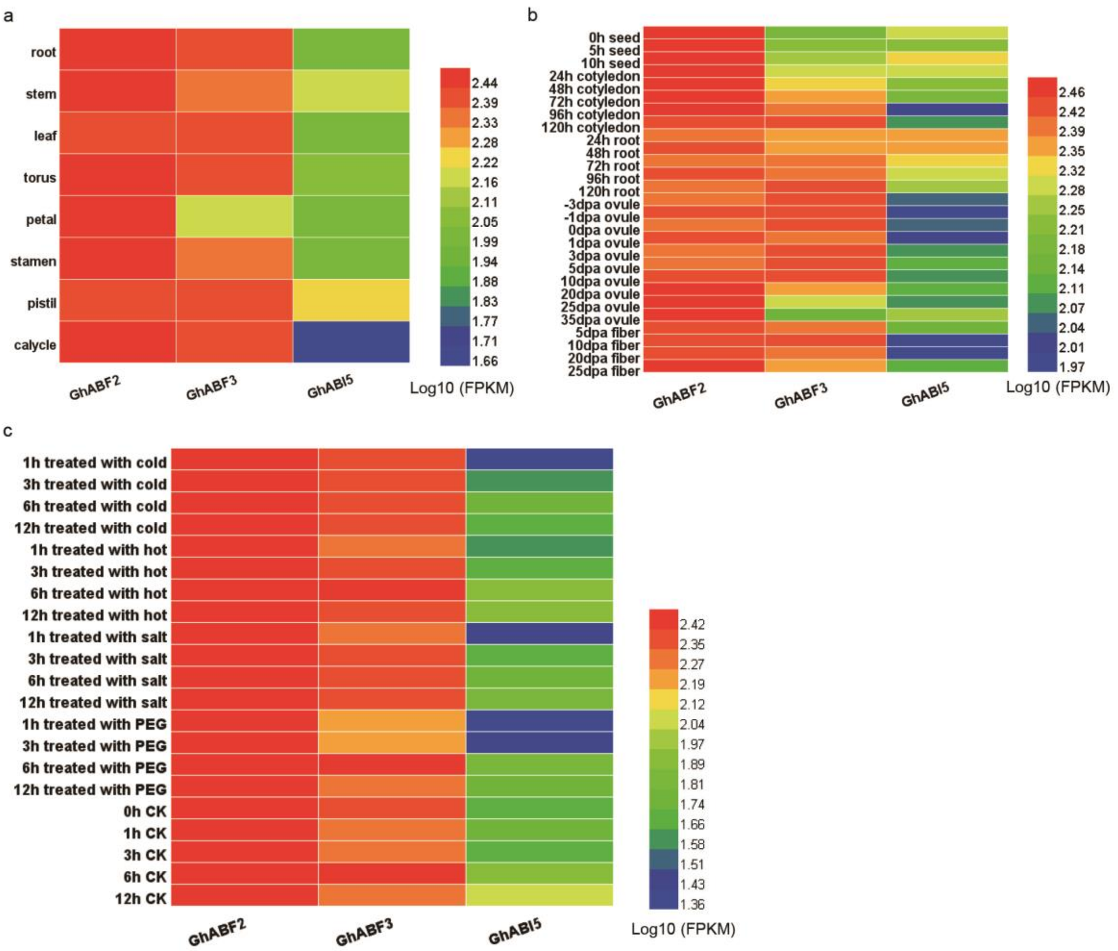

Figure S3. Expression pattern of *GhABF2*, *GhABF3*, and *GhABI5*. The data were extracted from NCBI (http://www.ncbi.nlm.nih.gov/bioproject/PRJNA248163/).

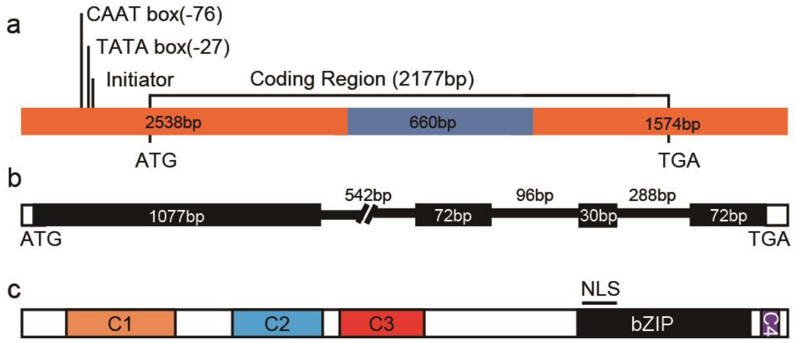

Figure S4. Cloning and characterization of *GhABF2* and subcellular localization of the GhABF2-GFP fusion protein.

(a) Schematic representation of genome region of GhABF2 isolated by TAIL-PCR and 3’-RACE PCR. Blue box represent DNA sequence obtained using degenerate primers. Orange boxes represent flanking regions.

(b) Structure of *GhABF2* coding region. The white boxes represent the 5’ and 3’ untranslated regions, the black boxes represent the coding regions and line between boxes represent introns.

(c) Structure of GhABF2 protein. C1, C2, C3, and C4 represent the conserved regions. NLS, nuclear localization signal.

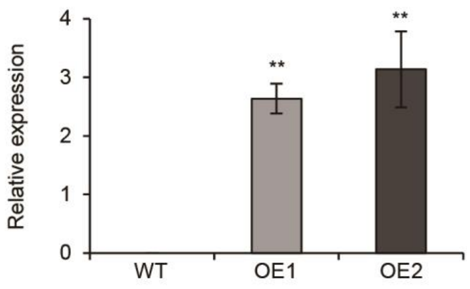

Figure S5. Transcript level of *GhABF2* in overexpression transgenic *Arabidopsis*. **P ≤0.01; Student t test.

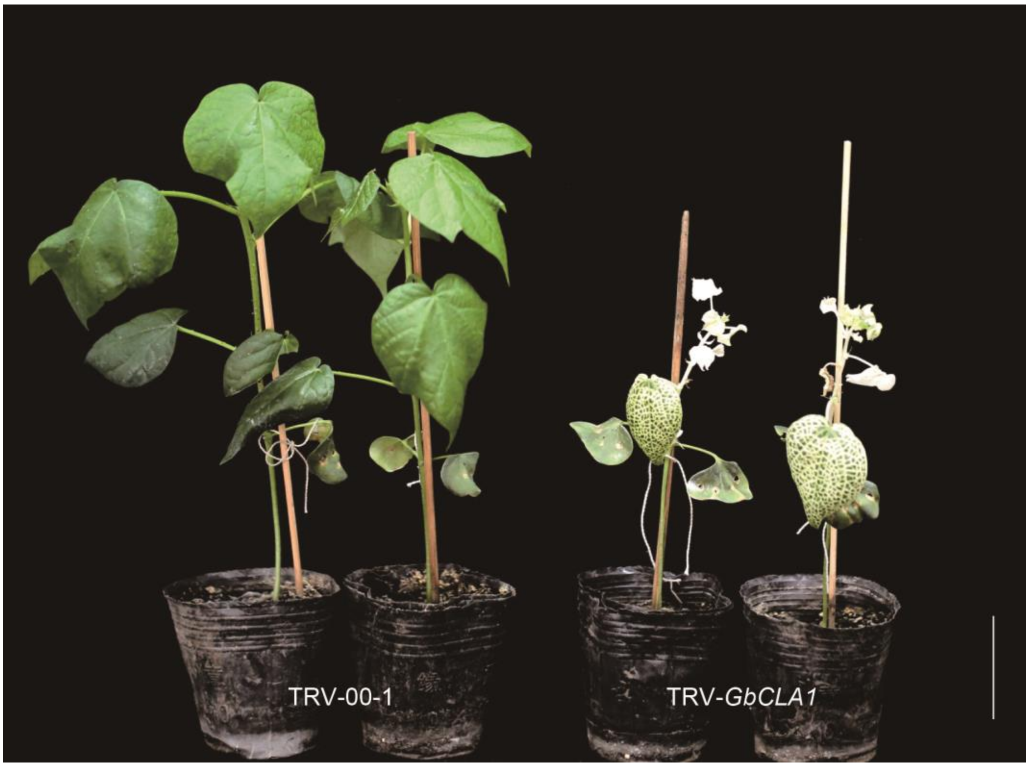

Figure S6. Silencing of the endogenous *G.barbadense cloroplastos alterados 1* gene in cotton by tobacco rattle virus (TRV)-mediated virus-induced gene silencing (VIGUS). TRV-00, VIGS-vector control. The photobleaching phenotype was photographed at three weeks post-infiltration. Bar = 5 cm.

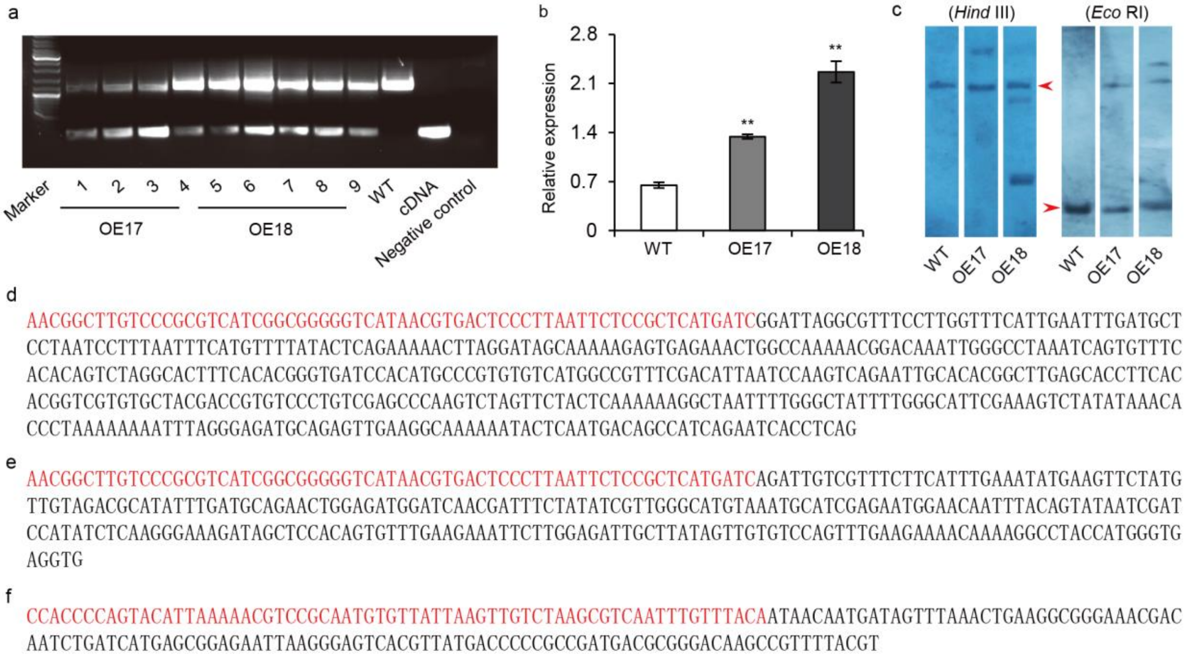

Figure S7. (a) The transgenic plants were detected by PCR. Genomic sequence (up, 1,000 base pair), cDNA sequence (down, 250 base pair). (b) Expression of *GhABF2* in overexpression transgenic lines. ***P* ≤0.01; Student *t* test. (c) Screening of transgenic cotton plants with targeted insertion using Southern blotting. Genomic DNA was digested by *Hind*III (left) and *Eco* RI (right). The red arrow indicates the endogenous gene of *GhABF2*. (d) Flanking sequence of OE17 cotton. (e and f) Flanking sequences of OE18 cotton. The red character indicates the vector sequence.

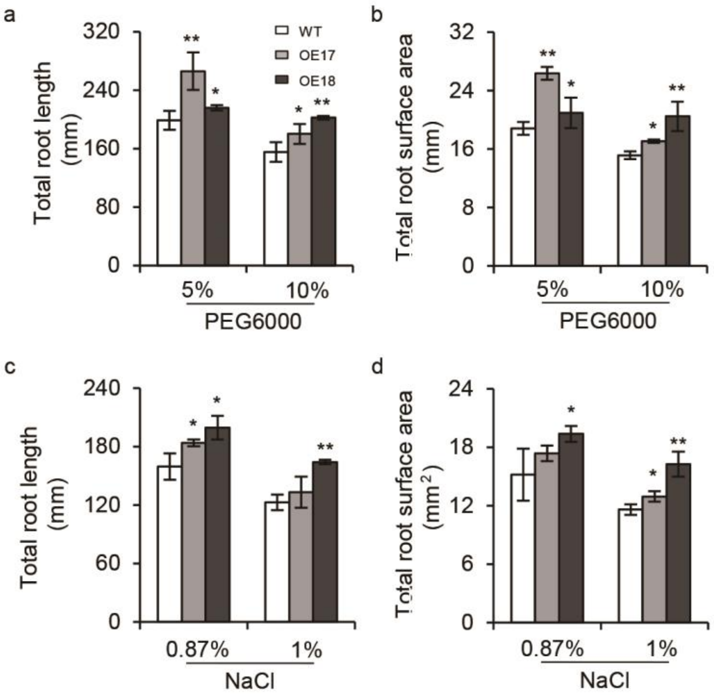

Figure S8. Total root surface area (a) and total root length (b) corresponding to Fig4a-b. Total root surface area (c) and total root length (d) under salt treatment. *P ≤0.01, **P ≤0.01; Student t test.

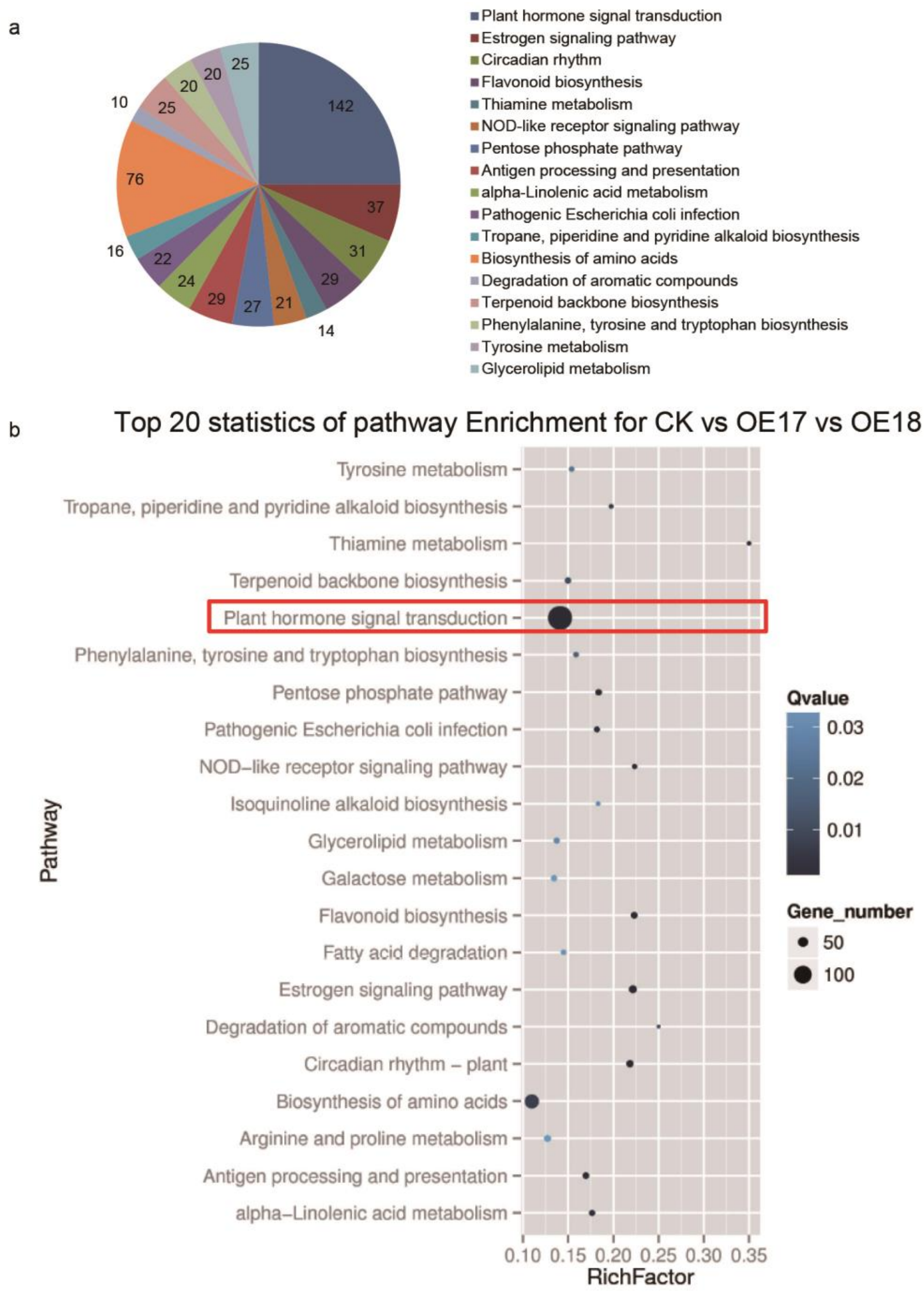

Figure S9. (a) Enriched DEG numbers from control vs OE17 and OE18 with annotation in known pathways. (b) DEGs enriched “plant hormone signaling pathway” as the top annotated pathway as marked by the red box.

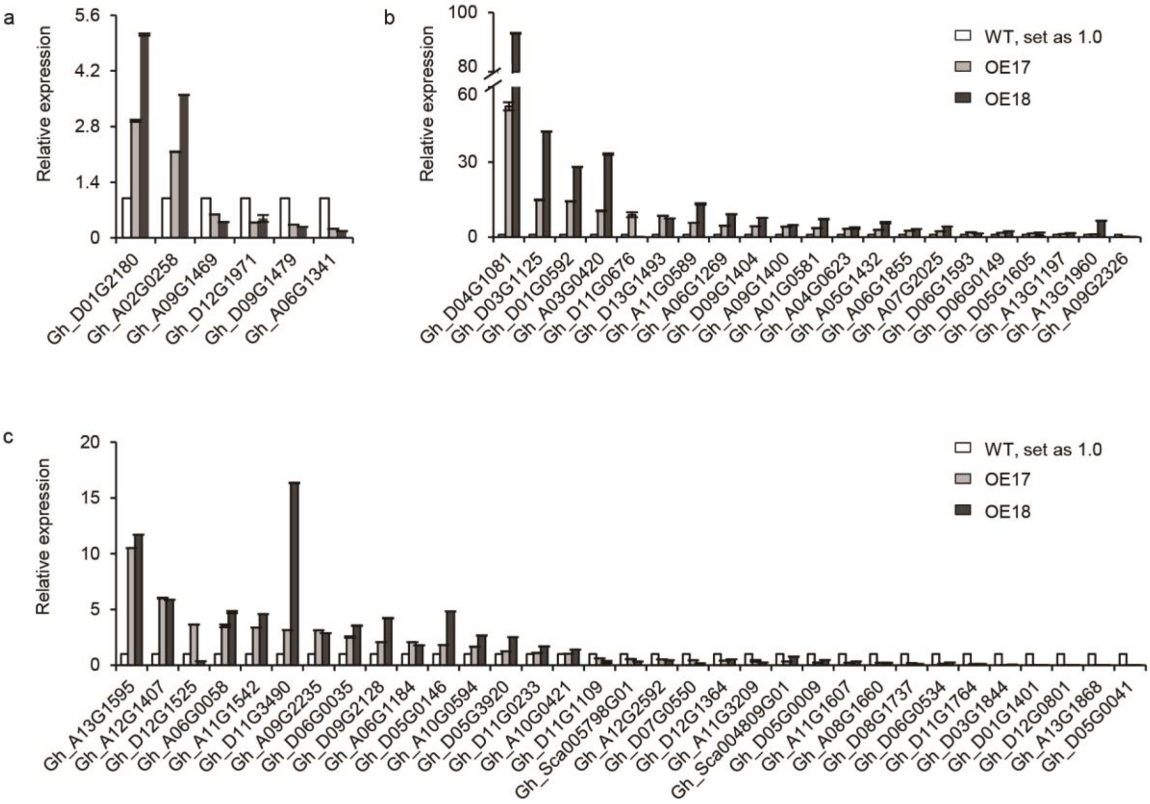

Figure S10. Expression of OR genes (a), CB genes (b) and others (c) by qRT-PCR. OR, oxidation reduction. CB, chlorophyll biosynthetic. Values are means ± SD of three replicates.

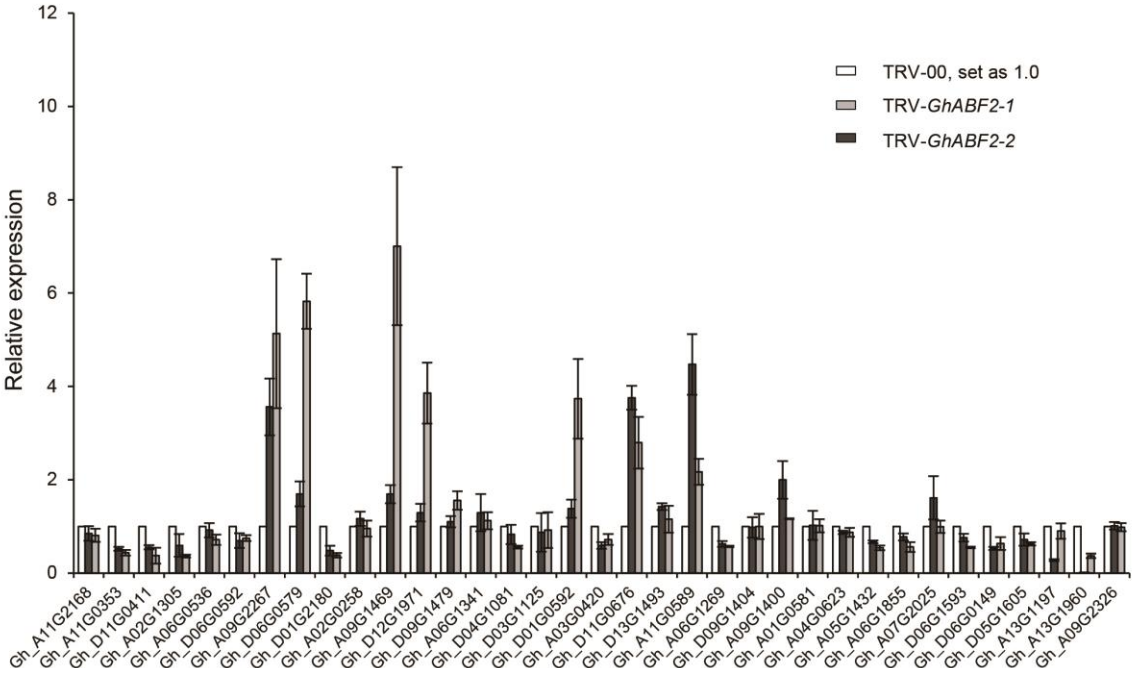

Figure S11. Expression of DEGs in TRV-00 and TRV-*GhABF2* cottons by qRT-PCR. Values are means ±SD of three replicates.

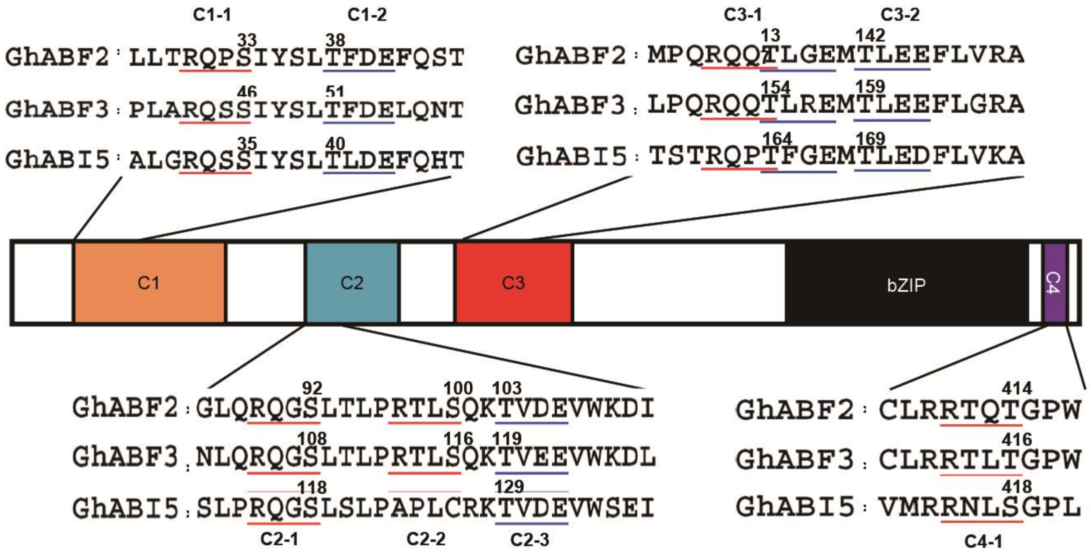

Figure S12. Like *Arabidopsis*, the conserved regions, C1, C2, C3, and C4 contain Putative protein kinase target sequences. In the conserved regions of GhABF2, GhABF3, and GhABI5, the most common sequences were R-X-X-ST (C1-1, C2-12, C3-1, and C4-1, putative targets for CDPK etc.) and ST-X-X-ED (C1-2, C2-3, C3-1, and C3-2, for CK II).

Table S1. Primers used in this study.

	Forward Primers	Reverse Primers
**Primers Used for Tail-PCR**		
GhABF2SP1	CCTCCATTTCCCACTAGGCCAACAC	
GhABF2SP1	CTTTGCCTATTCCACCCATTGTGC	
GhABF2SP1	GGTTTATTACCACCGCCGCCATCAC	
AD1	NTCGASTWTSGWGTT	
AD2	NGTCGASWGANAWGAA	
AD3	WGTGNAGWANCANAGA	
AD4	GWGNAGWANCASAGA	
**Primers Used for Cloning of GhABF2**		
bZIPADP1	TGGARGAGTTTYTRGTYAGAGC	GCYTGTTTTCTKGCTCTAGATC
bZIPADP2	TKGAGGATTTCTTGGTKARGC	GCCTGCTTSCGDGCCCKTGAYC
bZIPFP1	AWGATHAARAAYMGVGARTCHGCDGCDMGDTCHMGVGC	
bZIPFP2	GARTCHGCDGCDMGDTCHMGVGCHMGVARRCARGCWYAH	
AMV OligoP	GCTGTCAACGATACGCTACGTAACGGCATGACAGTGTTTTTTTTTTTTTTTTT	
RACERVP1	GCTGTCAACGATACGCTACGTAACG	
RACERVP2	CGCTACGTAACGGCATGACAGTG	
GhABF2ORFP	ATTTCATTTAGAAAACTGGGTTTTCTTTTGAATAATTAATCGACTC	
GhABF2RV1	CTTCCCAATCCCATCTGACG	
GhABF2RV2	CCTTGCAGTGCACCTGCCTG	
GhABFORF	ATGGGGACTAACATGAACTT	CCAAGGACCGGTCTGGGTTC
**Primers for probe amplification for Southern hybridization**		
GhABF2	GTAACCCACCGCCGTCAGGTG	TCCCTGATCCCCAATCCCATGC
**Primers Used to RT-PCR and qRT-PCR**		
GhABF2	TCCAAATTTGATGGGAACTAG	CTGCTTCCAATTCCATTGTAT
GhACT7	CTCTCTCTGTATGCCAGTGGTC	TTGTCCGTCAGGCAACTCATAG
NPTII	GGAGTGAAAGAGCCTGATGC	CGGCTCCGTCGATACTATGT

Gh_A01G0581	AGGGAGAGTTGGTGGTGTTG	TCCCAATGCTTTTCAACCTC
Gh_A02G0258	GAGGAAAGCCAATTGCAGAG	CCATGTTCCTCAATCGTCCT
Gh_A02G1305	TCAGTTGCTGCCTTCCTTTT	CAAGGTGGAAAGGGTTGAAA
Gh_A03G0420	GCAAAACGGATGACAGGAAT	TACGAGTCAGTTCCCCTTGG
Gh_A04G0623	ACTTGAAGGGAGAGCGTTGA	CGAAACCCCTTGATCTACCA
Gh_A05G1432	ACAAACTGGGCTTTGGTGTC	TTCATTCCAGGGACGCTATC
Gh_A06G0058	ACCAAGGTCGGTGATGAGAC	GGGGACTGTGTTGACAGCTT
Gh_A06G0536	GAAGACCCTGACCTCCTTCC	TGGGGACGAAATTACTCCTG
Gh_A06G1184	GAATGTTTTTCACCGCCACT	GCTTCAATGTACCCCCTTCA
Gh_A06G1269	TCGTCCTCACACAACTCTGC	TGGGGTTTTTGCTAATCTCG
Gh_A06G1341	GCTGGCTGAGAAACTTTTGG	GCCCTTAGCCCCTTGATTAG
Gh_A06G1855	GAGGAAGCCGAGTACAGTGC	TGGATGGTAAGGAAGCAACC
Gh_A07G2025	ACCACGAGGAAGAAGAAGCA	GCGTTTGAGAGCATGAACAA
Gh_A08G1660	CGCAGCAGTTGACTGAACAT	GTGGCTGCTTCTAGGCAAAC
Gh_A09G1400	TGCAACATCTGCCTCATCTC	AGGAACCCGGAAGAAAAAGA
Gh_A09G1469	CTCTTGGATGTCACCCCACT	CACCTGGATCAACACACCAG
Gh_A09G2235	GCCTGTGCTTCTCTTGAACC	GCCGACTTGTTCCCTGAATA
Gh_A09G2267	AGGTGGCTGATTACCACAGG	GCAGAAGGGACAATGGTGTT
Gh_A09G2326	TTCGAGAAAACCCAACCAAC	CCCTTCCGCATTGGTAACTA
Gh_A10G0421	GCCTCCAAAAGCTTCCTTCT	TGTAGCAGCCTTCCCGTTAT
Gh_A10G0594	CTTGTGGTGGGCAGAAACTT	AGGCCCTTTGAATCTGGTTT
Gh_A11G0353	GCAATGACGGCTTGAAAAAT	CATAAGGGCCTTTCCAGTCA
Gh_A11G0589	CGACCCTTCAATGGTTGACT	AACCGTTTTGGCATCATTGT
Gh_A11G1542	CTCAAGGTCACATCCCGAAT	ATCAGCTAGGGTTCGAGCAA
Gh_A11G1607	GTCGTTTCTGGGGAAGAACA	AAACCATCAACCAGGCTCAC
Gh_A11G2168	CATTAAAAGCCCTCCAACCA	CTCAACTGGGGAAGCTTTTG
Gh_A11G3209	TGTGCAGACCCTGCTATGAG	AAACCACGGCCAGTGTTAAG
Gh_A12G1407	ACACCAAGGGATGTTGCTTC	GCCTCGAGAGTGGTCAGTTC
Gh_A12G2592	TGAAGCAATGGCTGAAAGTG	AGCCAATGTTGCCAGTAACC
Gh_A13G1197	TCTCCAATTTCCCACGAAAC	CCAAACTCTCTCCCCATCAA
Gh_A13G1595	ATCACCTTACCGAGCACCAC	GGTGGAATCAGAAGCAGCTC
Gh_A13G1868	CGGTTTTATCTCACCGGAAA	CCGTGAGACGGTTCAATTTT
Gh_D05G3920	TGGAATCGGGAGAATTGAAG	CTCGTATAACGGGTGGGCTA
Gh_D06G0035	TCCTCAAGGTGGTCATCTCC	ACCGTGTAGCCCATCTTGTC
Gh_D06G0149	GAGGAAGCCGAGTACAGTGC	TGGATGGTAAGGAAGCAACC
Gh_D06G0534	TTCCAAATTCCTGCAGGTTC	TCGAGCCACTTCCAGTCTTT
Gh_D06G0579	TCTAAACCCAAGGCTCAGGA	TCCCTTCGTCAAGCTTCCTA
Gh_D06G0592	GGATCCCGATAAGTGGGAAT	TCATCCCTTGCAGCTCTTCT

Gh_D06G1593	CTGGAATGAAGGCACTGGAT	GCTGTACCCTTTCAGCTTCG
Gh_D07G0550	GATGATGATCCCGTGAATCC	CACTAGCACCTCTGCCATCA
Gh_D08G1737	CCAGAATCGACTGGAAGGAA	CCATGGAAGCTTTCACCTGT
Gh_D09G1404	TCCGACTCAACTCCCAATTC	TCATCATCGCTGCCAAATAA
Gh_D09G1479	GCTTATGGTGCTGCAGTTCA	CACCTGGATCAACACACCAG
Gh_D09G2128	GCCTGTGCTTCTCTTGAACC	GCCGACTTGTTCCCTGAATA
Gh_D11G0233	ACTGAGCCATGACACAACCA	CTCCTTCTCGGGCTTCTTCT
Gh_D11G0411	TGACTGGAAAGGCCCTTATG	TTCAGTACTGCTCGCCCTTT
Gh_D11G0676	CACCACCTACGACACTTCCA	AACCGTTTTGGCATCATTGT
Gh_D11G1109	GAGAGTGAAGCGGGTAGTGC	CACCAGGGCTAGAGATACGC
Gh_D11G1764	TGCCTACAGCAGTCCAACAG	GGGAATTGGGACAGAATGTG
Gh_D11G3490	ACGAGCCAGATGCAGAGACT	ATTCGGGATGTGACCTTGAG
Gh_D12G0801	TGTCCAGTCAACTGCTGTGC	GACCCGACTTGGGTGAAACT
Gh_D12G1364	CCCATTCCCTTCTTCACTCA	GATTTGAAGCACCCTGTCGT
Gh_D12G1525	ACACCAAGGGATGTTGCTTC	GCCTCGAGAGTGGTCAGTTC
Gh_D12G1971	AAGAGGCTCCTCTCATGCAA	CATGTTCAGGACTTGGCTCA
Gh_D13G1493	TCTCCAATCTCCCACGAAAC	CCAAACTCTCTCCCCATCAA
Gh_Sca004809G01	GGCACTCTTTGCCTTGACTC	TTGCTCCCAAGTAACCCAAC
Gh_Sca005798G01	CAAAACAGCAACTCGACCAA	TACCACCAGTGACAGGCAAA
**Primers Used for Vector Construction**		
GhABF2ORFFP	GACTGCAGGATGGGGACTAACTGAACTTTG	CCCTCGAGTCACCAAGGACCGGTCTGGGTTC
Note	M: A or C, K: G or T, H: A or C or T, R: A or G, Y: C or T, D: A or G or T	
